# Supplementary figures and images for: Full-Length Transcriptome Analysis of the Ichthyotoxic Harmful Alga Heterosigma akashiwo (Raphidophyceae) Using Single-Molecule Real-Time Sequencing
Source: Microorganisms. 2023 Feb 3;11(2):389. doi: 10.3390/microorganisms11020389 (PMC9959365; doi:10.3390/microorganisms11020389)

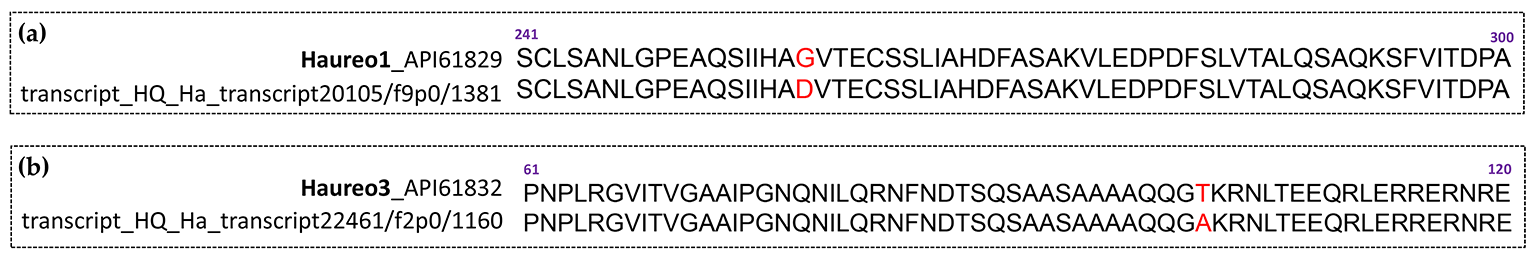

Supplement: Supplementary file 1 [file microorganisms-11-00389-s001.zip › Supplementary materials/Figure S1.tif]
